# Building Australia’s non-animal technologies ecosystem: a national cross-sector mapping and strategic insights from the non-animal technologies network (NAT-Net)

**DOI:** 10.3389/ftox.2026.1790806

**Published:** 2026-03-27

**Authors:** Shafagh A. Waters, Adam P. Hill, Esther S. Pronker, Gerard E. Kaiko, Gungun Lin, Simon Keely, Laura Collie, Wojciech Chrzanowski, Anai Gonzalez-Cordero

**Affiliations:** 1 School of Biomedical Sciences, Faculty of Medicine & Health, University of New South Wales, Sydney, NSW, Australia; 2 Non-Animal Technologies Network (NAT-Net), University of New South Wales, Sydney, NSW, Australia; 3 Department of Respiratory Medicine, Sydney Children’s Hospital, Randwick, NSW, Australia; 4 Victor Chang Cardiac Research Institute, Darlinghurst, NSW, Australia; 5 School of Clinical Medicine, University of New South Wales, Sydney, NSW, Australia; 6 School of Biomedical Sciences and Pharmacy, College of Health, Medicine and Wellbeing, University of Newcastle, Callaghan, NSW, Australia; 7 Institute for Biomedical Materials & Devices, School of Mathematical and Physical Sciences, Faculty of Science, University of Technology Sydney, Sydney, NSW, Australia; 8 Immune Health Research Program, Hunter Medical Research Institute, New Lambton Heights, NSW, Australia; 9 Office for Health and Medical Research, NSW Ministry of Health, Sydney, NSW, Australia; 10 School of Population Health, Faculty of Medicine & Health, University of New South Wales, Sydney, NSW, Australia; 11 Sydney Pharmacy School, Faculty of Medicine & Health, The University of Sydney, Camperdown, NSW, Australia; 12 Division of Biomedical Engineering, Department of Materials Science and Engineering, Uppsala University, Uppsala, Sweden; 13 Stem Cell Medicine Unit, Children’s Medical Research Institute, Westmead, NSW, Australia; 14 School of Medical Sciences, Faculty of Medicine & Health, The University of Sydney, Sydney, NSW, Australia

**Keywords:** 3Rs, Australia, in silico models, microphysiologicalsystems, network, new approach methodologies, non-animal technologies, organoids

## Abstract

**Background:**

Non-animal technologies (NATs; also termed new approach methodologies, NAMs) are rapidly reshaping biomedical research, safety testing and drug development by improving human relevance and reducing reliance on animal models. In Australia, substantial expertise exists across organoids, microphysiological systems and *in silico* approaches; however, national visibility of capabilities, infrastructure access and system-level needs has been limited.

**Methods:**

We conducted a cross-sectional, online baseline survey (February–October 2025; https://redcap.ohmr.health.nsw.gov.au/surveys/?s=3EWRM48PEXWLFJC7.) integrated into NAT-Net registration and implemented using REDCap. The survey captured organisational characteristics, roles within the NAT ecosystem, research domains, model types, development stage, supporting services and infrastructure access, engagement preferences, and perceived challenges. Quantitative data were summarised descriptively, and free-text responses were analysed thematically.

**Results:**

Of 135 submitted records, incomplete entries (n = 2) and duplicates (n = 9) were removed, yielding 124 unique respondents. Respondents were predominantly based in New South Wales (71.0%), followed by Victoria (18.5%) and Queensland (7.3%). Most organisations reported involvement in research and development (79.0%), education and training (41.9%), ethics and the 3Rs (39.5%), and provision of supporting services or facilities (32.3%). Among respondents engaged in NAT R&D (n = 102), commonly reported approaches included *in vitro* 3D models (64.7%), *in vitro* 2D culture (56.9%), microphysiological systems/organ-on-chip platforms (35.3%), and *in silico* methods (30.4%). Among service providers answering the access item (n = 40), 60.0% offered external access. The most frequently identified challenges were the absence of standards and validation processes (75.8%), limited access to resources and infrastructure (74.2%), and difficulty in establishing collaborations or partnerships (71.7%).

**Conclusion:**

This NAT-Net baseline survey provides an initial cross-sector ecosystem mapping centred on NAT-Net participants, revealing broad cross-sectoral activity alongside persistent system-level barriers to standardisation, infrastructure access and coordination. These findings provide a critical evidence base to inform coordinated national strategy, investment, policy development and longitudinal monitoring.

## Graphical Abstract

**Figure:**
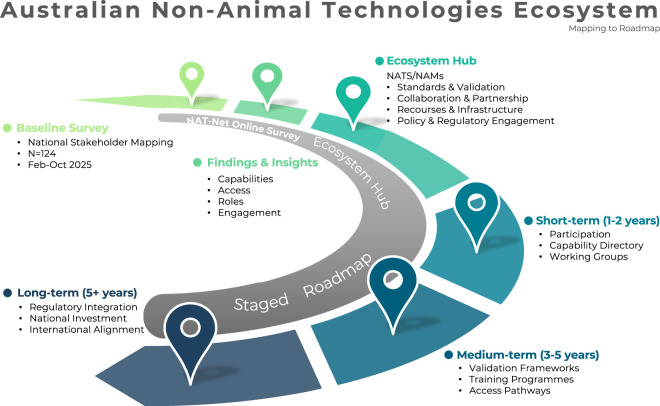

The NAT-Net baseline survey was used to map the distribution of non-animal technology capabilities, infrastructure, organisational roles and engagement preferences across Australia. Survey findings revealed key system-level barriers, including limited standards and validation processes, restricted infrastructure access, and challenges in forming collaborations and partnerships. The NAT-Net network functions as an ecosystem coordination hub, enabling strategic actions across standards and validation, access to resources and infrastructure, collaboration and partnerships, training and workforce development, and policy and regulatory engagement. These coordinated mechanisms aim to accelerate national uptake of non-animal technologies, improve human-relevance in biomedical research and testing, and reduce reliance on animal use.

## Introduction

Non-animal technologies (NATs), also referred to as new approach methodologies (NAMs), have emerged as a transformative class of experimental platforms designed to complement or replace the use of animals in research, drug development and safety testing, as well as in medical/veterinary education. While animal models have long underpinned biomedical discovery and regulatory decision-making, their limitations—including incomplete human translatability, high cost, long development timelines, and ethical concerns—have become increasingly apparent (Bracken, 2009). These constraints, coupled with advances in human cell biology, bioengineering and computational science, have driven a global shift toward more human-relevant, mechanistically precise experimental systems.

NATs encompass a broad spectrum of approaches, including *in vitro* and *in chemico* methods as well as *in silico* strategies. These include human stem cell-derived organoids, complex three-dimensional co-culture systems, microphysiological systems and organ-on-chip platforms, bioprinting technologies, and *in silico* approaches including computational modelling and artificial intelligence/machine learning (AI/ML). AI/ML also increasingly acts as an enabling layer across experimental NAT platforms (e.g., image-based phenotyping, multi-omics integration and organ-on-chip readouts). Collectively, these approaches enable interrogation of human biology in controlled, scalable, age-appropriate, and disease-relevant contexts, offering significant advantages for disease modelling, drug discovery, efficacy and toxicity testing, and personalised medicine. In some contexts, they also support early-stage regenerative and cell-based therapeutic development. However, despite their technical maturity and growing validation base demonstrating improved prediction of human biological and toxicological responses, widespread integration of NATs into routine research practice and regulatory decision-making remains uneven and often constrained by fragmented infrastructure, limited standardisation, and inconsistent stakeholder confidence.

Internationally, regulatory agencies and funding bodies are actively advancing frameworks to accelerate the development, validation, and adoption of NATs. The European Medicines Agency and associated European initiatives have established coordinated programmes to reduce reliance on animal testing (European Medicines Agency, 2024) and facilitate regulatory acceptance of NAMs. In the United States, the FDA Modernization Act 3.0 (Buntz, 2025) and subsequent initiatives, including the NIH Common Fund Complement-ARIE (National Institutes of Health, 2023) programme, have catalysed substantial investment in human-relevant methods. In the United Kingdom, government and regulatory bodies have formally announced a strategic shift toward the adoption of NAMs, supported by dedicated funding, infrastructure initiatives and cross-sector partnership (UK Government, 2025). Collectively, these efforts reflect a broader global trend toward structured ecosystem development—linking technology developers, end-users, regulators, and policymakers—and emphasise the importance of mapping capability, understanding strengths and limitations, harmonising standards where appropriate, and enabling regulatory qualification.

In Australia, significant expertise exists across organoid biology, microphysiological systems, computational modelling and enabling infrastructure. However, this capability has historically been distributed across institutions, jurisdictions, and sectors, with limited national visibility of where expertise resides, how infrastructure can be accessed, and what barriers constrain translation away from animal-based models. Globally, this landscape is rapidly changing, with coordinated regulatory, policy and funding initiatives emerging across Europe, the United Kingdom, North America and Asia to accelerate the development, validation and implementation of non-animal approaches (European Commission, 2023; European Medicines Agency, 2022; UK Government, 2025, Government of Canada, 2025; Jang, 2025; Organisation for Economic Co-operation and Development, 2025). Furthermore, in stark contrast to the EU, USA, UK, and other countries, Australia lacks dedicated national funding mechanisms and coordinated infrastructure strategies specifically targeting the development and implementation of non-animal technologies. This fragmentation has impeded systematic knowledge sharing, duplication minimisation, and coherent engagement with regulatory and policy stakeholders.

To address these gaps, the Non-Animal Technologies Network (NAT-Net) was established in 2024 with support from NSW Health as a state-initiated coordination platform with a national outlook to connect researchers, infrastructure providers, industry, regulators, government, and community stakeholders (NSW Health, 2025). NAT-Net was conceived to facilitate cross-sector collaboration, accelerate development and uptake of innovative non-animal models, enable training and workforce development, and support dialogue across the research-to-regulation pipeline. By embedding ecosystem mapping within its recruitment process, NAT-Net is structured to maintain a living, continuously evolving ecosystem map derived from participating stakeholders, with the potential to expand toward a broader national monitoring resource as network participation grows. Importantly, NAT-Net is positioned not merely as a networking platform, but as an ecosystem coordination hub, with a vision to support broader Australian alignment and coordination in this space, providing an evidence-informed understanding of national capability, infrastructure access, regulatory pathways, and strategic needs.

Effective coordination and policy development require an accurate baseline understanding of the existing ecosystem: who is engaged, where they are located, what technologies and services are represented, how mature these approaches are, and what challenges and priorities stakeholders perceive. To this end, we designed and implemented a national baseline survey integrated into NAT-Net registration, capturing NAT-related activities, capabilities, infrastructure and priorities across Australia and Aotearoa New Zealand. The objectives of this study were to: (1) characterise the organisations, roles and jurisdictions currently engaged in NATs; (2) describe the breadth of research domains, model types and development stages represented; (3) map existing NAT-related supporting services and infrastructure; (4) document preferred modes of engagement with NAT-Net; and (5) identify perceived challenges and gaps that a coordinated national network could help address. Here, we report descriptive findings from the NAT-Net baseline survey and discuss their implications for national strategy, coordination, and policy development in non-animal technologies.

## Materials and methods

### Study design and setting

We conducted a cross-sectional, online survey implemented in REDCap (hosted by the NSW Office for Health and Medical Research). The instrument served both as NAT-Net membership registration and as a structured mapping tool to characterise NAT-related activities, capabilities and needs across Australia and Aotearoa New Zealand. A small number of respondents were based in Aotearoa New Zealand; these were retained in all analyses but were too few to support separate statistical comparison and do not materially influence overall conclusions. Reporting follows the STROBE guidelines (Jan P. Vandenbroucke et al., 2007) for cross-sectional studies, including explicit description of sampling, response flow, variables, and analysis.

### Survey development and content

The NAT-Net registration and mapping instrument was developed by the NAT-Net Executive Committee and iteratively refined through internal consultation with representatives from academia, infrastructure, ethics and government. Pilot testing with anticipated users assessed clarity, wording, response options, survey length and branching logic; minor edits were made to reduce ambiguity and respondent burden. The REDCap instrument used branching logic to display sections relevant to respondents’ roles (e.g., R&D-only modules; infrastructure-only modules). Core registration items were mandatory, while most technical mapping items were optional to accommodate the diversity of stakeholder roles.

The final instrument comprised three sections: (1) About you and your expertise (organisation type and jurisdiction; roles in the NAT ecosystem; plus, R&D-specific domains, model types and development stage; infrastructure-specific access and fee structures); (2) Share your thoughts (engagement preferences and perceived challenges/gaps, including free-text responses); and (3) Stay connected (opt-in contact preferences). Multiple-response items permitted the selection of more than one option.

### Participants and recruitment

Eligible participants were individuals working in organisations engaged in NAT-related activities (including research, infrastructure, commercialisation, policy, ethics/3Rs, funding, advocacy, and education), as well as members of the public, media and advocacy groups with an interest in non-animal technologies. The survey link was disseminated through NAT-Net partners, mailing lists, professional societies, events and presentations, and informal forwarding. Because dissemination occurred through open network distribution and informal forwarding rather than a defined sampling frame, the total number of individuals who received the survey invitation is unknown. Consequently, a conventional response rate cannot be calculated, and the sample should not be interpreted as representative of all stakeholders engaged in non-animal technologies. The survey remains open; data presented here encompass responses submitted between February and October 2025.

### Data handling, privacy, and variables

Personal identifiers were stored separately from analytic survey responses in access-restricted datasets and were not used in analysis. We report aggregated, de-identified results only. Respondents’ opt-in communication preferences were stored with identifiers and were not linked to the analytic dataset. The survey captured high-level capability and needs information and did not solicit IP-sensitive protocols, proprietary methods, or confidential commercial details.

Key variables included jurisdiction, organisation type, roles, and activities within the NAT ecosystem (multi-response), R&D domains, model types, development stage, supporting services and infrastructure, access arrangements, engagement preferences, and perceived challenges or gaps. Free text “other” responses were reviewed and mapped to existing categories or grouped into descriptive themes.

Duplicate submissions were screened prior to analysis. Because survey registration captured identifiable respondent information (e.g., name and/or email), duplicate entries were identified through manual verification of respondent identity and removed before de-identification of the dataset. Multiple respondents from the same organisation were retained, as the unit of analysis was the individual stakeholder perspective rather than organisational representation. The dataset therefore reflects diversity of views within organisations and sectors rather than institutional counts.

### Statistical and qualitative analysis

Descriptive statistics (counts and percentages) were generated for quantitative items. For multi-response questions, percentages were calculated using the number of respondents who answered that item as the denominator; totals may exceed 100%. Free-text responses were thematically grouped by two members of the steering group, with discrepancies resolved by discussion.

A small number of exploratory, hypothesis-generating within-sample comparisons were conducted using Fisher’s exact tests for selected binary variables (excluding jurisdiction-based comparisons due to convenience sampling and geographic skew). These analyses were not adjusted for multiple comparisons and are interpreted cautiously. They are intended only to generate hypotheses within the respondent sample and are not generalisable to the broader national ecosystem.

### Ethics statement

Participation in the NAT-Net baseline survey was voluntary, and respondents could discontinue at any time. An information statement provided at survey entry described the purpose of the activity, the voluntary nature of participation, and data handling procedures; consent was implied by survey completion.

The activity was reviewed by the NSW Office for Health and Medical Research (OHMR) Research Ethics and Governance Office and based on advice from the OHMR Manager of Research Ethics and Governance, was determined not to constitute human research under the National Statement on Ethical Conduct in Human Research (NHMRC) and therefore did not require review by a Human Research Ethics Committee (HREC). The survey was classified as out of scope for HREC review and did not meet NHMRC triggers for ethical review of quality assurance, evaluation, or non-research activities.

All data were collected and reported in a de-identified, aggregated form. No sensitive personal information, health records, biospecimens or clinical data were collected. Personal identifiers were stored separately from analytic survey data in access-restricted datasets and were not used in analysis.

## Results

### Response flow and respondent profile

**FIGURE 1:**
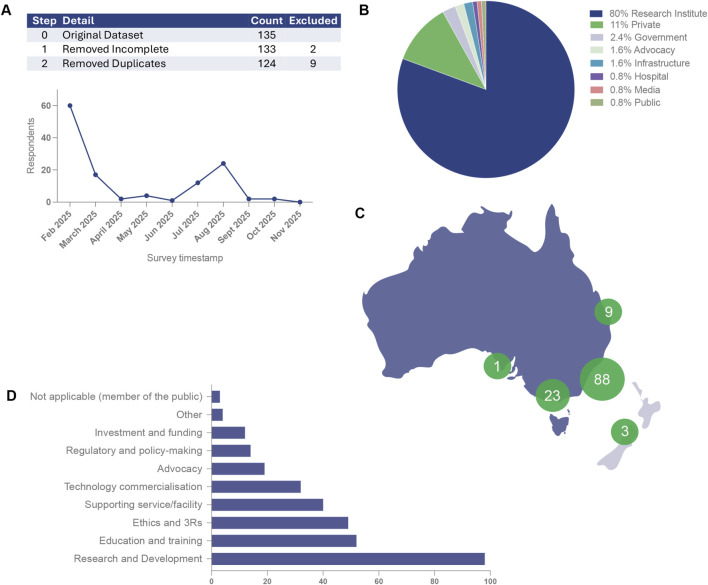
Survey response flow, respondent profile and geographic distribution for the NAT-Net baseline mapping survey. **(A)** Data cleaning workflow and response timeline showing the derivation of the final analytic sample. Of 135 submitted records, two incomplete responses and nine duplicate entries were excluded, yielding 124 unique respondents included in the final analysis. **(B)** Organisational profile of respondents, illustrating representation across universities and medical research institutes, national research infrastructure, hospitals, government agencies, industry/private companies, advocacy organisations, media and members of the public. **(C)** Geographic distribution of respondents by Australian state or territory and “Other”, demonstrating a predominant concentration in New South Wales (71.0%), followed by Victoria (18.5%) and Queensland (7.3%), with smaller representation from South Australia and other jurisdictions (including Aotearoa New Zealand (n = 2) and an ACT-based national role). **(D)** Primary organisational roles within the non-animal technologies (NAT) ecosystem (multiple responses permitted), showing highest engagement in research and development, education and training, ethics/3Rs activities, and provision of supporting services or facilities, alongside contributions to commercialisation, advocacy, regulatory/policy activities and investment/funding Percentages are calculated using the number of respondents answering each item as the denominator; totals may exceed 100% due to multiple-response design.

Of 135 submitted records, two incomplete entries and nine duplicates were removed, leaving 124 unique respondents for analysis (multiple respondents could originate from the same organisation) (Figure 1A). Respondents represented universities, medical research institutes, national research infrastructure, hospitals, government, industry/private companies, advocacy organisations, media, and members of the public (Figure 1B).

The survey encompassed stakeholders across Australia, with the majority of respondents based in New South Wales (88/124, 71.0%), followed by Victoria (23/124, 18.5%) and Queensland (9/124, 7.3%). Smaller representation was observed from South Australia (1/124, 0.8%) and “Other” locations (3/124, 2.4%), including Aotearoa New Zealand and an ACT-based national role; the number of New Zealand respondents was small and did not materially affect overall findings (Figure 1C).

### Organisational activities and roles in the NAT ecosystem

Respondents reported engagement across a broad range of activities within the NATs domain, with multiple selections permitted. The most frequently cited involvement was in research and development (98/124; 79.0%), followed by education and training (52/124; 41.9%), ethics and the 3Rs (49/124; 39.5%), and provision of supporting services or facilities (40/124; 32.3%). Additional areas of engagement included technology commercialisation (32/124; 25.8%), advocacy (19/124; 15.3%), regulatory or policy-making activities (14/124; 11.3%), and investment or funding (12/124; 9.7%). A small proportion of respondents (4/124; 3.2%) indicated uncertainty about their role or identified as potential users of NATs seeking further information on available options (Figure 1D).

### Research domains, model types and development stage

**FIGURE 2:**
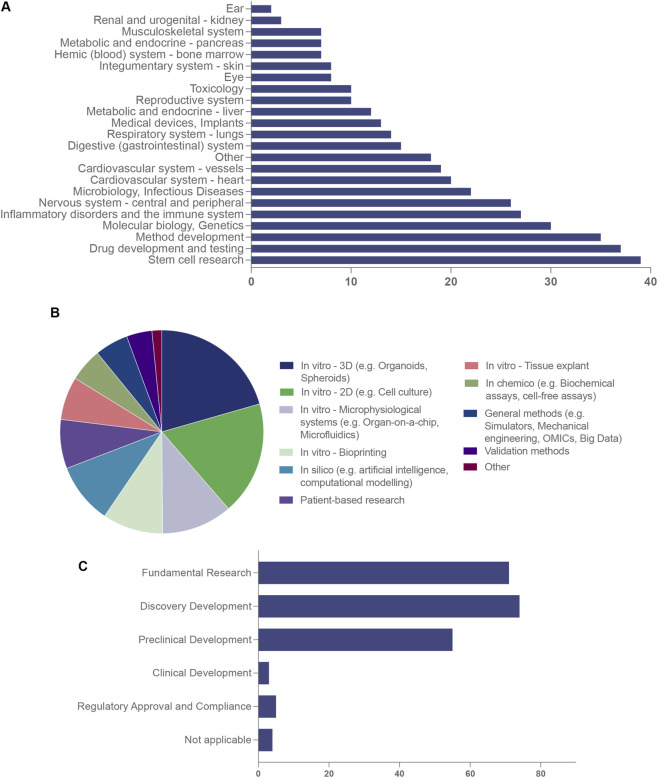
Research domains, model types and development stage represented among respondents engaged in non-animal technology research and development. **(A)** Broad scientific and disease domains in which NATs are applied (n = 102 R&D respondents; multiple responses permitted). Cross-cutting enabling areas such as stem cell research, drug development and testing, method development and molecular biology/genetics are prominently represented, alongside system- and disease-focused domains including immune/inflammatory, nervous system, cardiovascular, respiratory, metabolic/endocrine, and infectious disease research. Additional areas include medical devices and implants, big data and machine learning, advanced imaging, human behaviour change, synthetic model systems and *in vitro* platform technologies. **(B)** Types of non-animal models and methodological approaches reported by R&D respondents, spanning *in vitro* 3D models (e.g., organoids, spheroids and complex co-cultures), *in vitro* 2D cultures, microphysiological systems/organ-on-chip platforms, bioprinting approaches, *in silico* methods (including artificial intelligence and computational modelling), patient-derived research, tissue explants, *in chemico* assays, general methods (e.g., simulators, mechanical engineering, omics and big-data analytics) and validation strategies. **(C)** Self-reported development stage of NAT activities, indicating that most activity is concentrated at the fundamental, discovery and preclinical stages, with comparatively few respondents reporting clinical development or regulatory use, consistent with an ecosystem in which technical capability is well established but formal validation, qualification and regulatory integration are still emerging. Panels A and B reflect multi-response items; percentages may sum to >100%. Panel C reflects self-reported primary development stage.

Among respondents who reported their broad field of research related to NATs (n = 102/124), cross-cutting enabling domains were highly represented, including stem cell research, drug development and testing, method development, and molecular biology/genetics. Organ system- and disease-focused research were also prominent, with immune and inflammatory disorders reported by 27% of respondents, followed by nervous system (26%) and cardiovascular research (20%). Additional areas (18%) encompassed diverse topics including big data, machine learning and generative AI, cancer research, advanced imaging, respiratory and nasal epithelial studies, synthetic animal models, metabolic and endocrine research, human behaviour change, *in vitro* platform technologies, and stress metrics (Figure 2A).

Reported NAT model types encompassed both *in vitro* and computational approaches. Among respondents (n = 102), the most frequently cited were *in vitro* 3D models (66/102; 64.7%) and *in vitro* 2D culture systems (58/102; 56.9%). Organ-on-chip and other microphysiological systems were reported by 36 respondents (35.3%), while bioprinting and *in silico* methods were each reported by 31 respondents (30.4%). Additional approaches included patient-derived research, tissue explants, in chemico assays, general methodological frameworks, and validation strategies. A small proportion (approximately 5%) selected “Other” activities, encompassing strategic and economic analysis, chip-based technologies, human behaviour change research, analysis of human MRI data in adolescent populations, and the use of human biomarkers and wearable technologies (Figure 2B).

Most R&D activity was reported at the fundamental (basic mechanistic or platform development) (71/102; 69.6%), discovery (early target identification, screening, or proof-of-concept studies) (74/102; 72.5%), and preclinical (55/102; 53.9%) stages, with comparatively fewer respondents reporting clinical development (5/102; 4.9%) or regulatory use (3/102; 2.9%) (Figure 2C). When viewed along a simple NAT maturity continuum, these findings indicate that most Australian activity captured by the survey resides in early-stage model development and preclinical application, with relatively few respondents reporting use of NATs in clinical development or formal regulatory decision-making contexts. This pattern is consistent with an ecosystem in which technical capability is well established, but systematic validation, qualification, and regulatory integration of NATs are still emerging.

Additional R&D activities reported by respondents highlight emerging and enabling technologies within the NATs space. These include the development of complex organoids, such as pancreas organoids incorporating multiple cell types, and the integration of enabling technologies such as multiplexed microfluidic pumps, biosensors, fraction collectors, and advanced control systems. Respondents also noted the use of hydrogel-based phantoms and the development of chemically defined animal component-free alternatives to foetal bovine serum (FBS) for cell culture and cryopreservation. Importantly, these areas were not captured in earlier survey categories and represent additional dimensions of NATs innovation. Collectively, these initiatives underscore the breadth of innovation aimed at creating human-relevant models and infrastructure to accelerate NATs' adoption in Australia.

### Supporting services, infrastructure, and access

**FIGURE 3:**
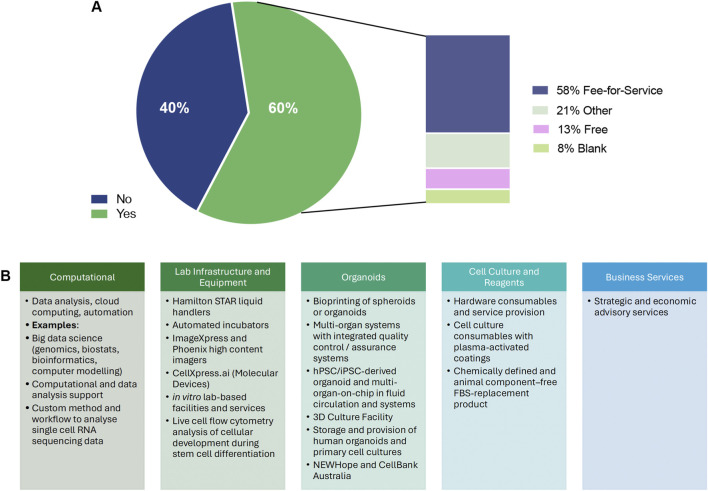
Availability, accessibility and types of supporting services and infrastructure for non-animal technologies in Australia. **(A)** Proportion of organisations providing NAT-related supporting services or facilities that offer external access (n = 40 service providers). Sixty percent (24/40) reported offering external access, while 40% (16/40) reported no external access. Among those offering access, mechanisms included fee-for-service, collaborative arrangements, licensing, service contracts and consortium-based models. **(B)** Categories of supporting services and infrastructure reported, including computational capabilities (data analysis, bioinformatics, cloud computing, automation and custom single-cell workflows), laboratory infrastructure and equipment (automated liquid handling, high-content imaging, *in vitro* facilities and live-cell flow cytometry), organoid and 3D culture resources (including bioprinting, multi-organ systems, hPSC/iPSC-derived platforms and biobanking support), specialised reagents and devices (e.g., plasma-activated coatings and chemically defined, animal component-free media), and business services (strategic and economic advisory support). Access and cost structures for facilities offering external use, illustrating the distribution of fee-for-service models, free access within collaborative or institutional arrangements, cost-recovery approaches, licensing models and hybrid access mechanisms. These data highlight a substantial but heterogeneously accessible national infrastructure base supporting the development and translation of non-animal technologies.

We next examined services and infrastructure available in Australia to support NATs' development. Forty respondents indicated that their organisations provided supporting services or facilities. Among those answering the access question (n = 40), 24 (60.0%) reported offering external access through mechanisms such as fee-for-service, collaboration, licensing, service contracts, or consortium-based arrangements, whereas 16 (40.0%) did not provide external access, citing limited capacity or services still under development (Figure 3A).

Reported services spanned multiple categories (Figure 3B), including computational capabilities (data analysis, cloud computing, big data science, bioinformatics, and custom workflows for single-cell RNA sequencing), laboratory infrastructure (automated liquid handlers, high-content imaging systems, *in vitro* facilities, and live-cell flow cytometry), and organoid-related resources (bioprinting, multi-organ systems, hPSC-derived organ-on-chip platforms, and 3D culture facilities with biobanking support). Additional offerings included cell culture and reagent services (hardware consumables, plasma-activated coatings, and chemically defined, animal component-free FBS replacements) as well as business services such as strategic and economic advisory support. Together, these resources highlight a substantial national infrastructure base for advancing NAT research and translation.

### Engagement preferences with NAT-Net

**FIGURE 4:**
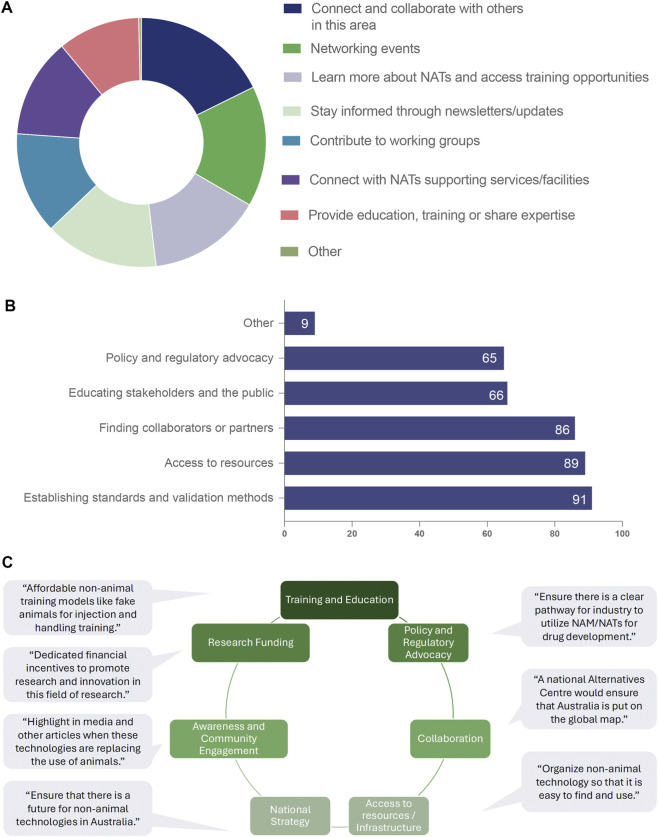
Engagement preferences, perceived challenges and thematic priorities for the NAT-Net community. **(A)** Preferred modes of engagement with NAT-Net among survey respondents (n = 124; multiple responses permitted), including connecting and collaborating with others, attending networking events, accessing training opportunities, receiving newsletters or updates, contributing to working groups, connecting with supporting services or facilities, and providing education, training or expertise. **(B)** Perceived challenges and gaps that NAT-Net could help address (n = 120 respondents), showing highest prioritisation of establishing standards and validation methods, improving access to resources, supporting the identification of collaborators or partners, educating stakeholders and the public, and policy and regulatory advocacy. Bars indicate the number of respondents selecting each option. **(C)** Thematic synthesis of free-text responses highlighting priority areas for NAT-Net action, including training and education, research funding, policy and regulatory advocacy, collaboration, access to resources and infrastructure, national strategy, and awareness and community engagement. Representative quotations illustrate stakeholder perspectives on workforce development, funding incentives, regulatory pathways, infrastructure coordination and national visibility of non-animal technologies. Panels A and B reflect multi-response items; percentages may sum to >100%. Panel **(C)** represents qualitative thematic grouping of free-text responses.

We next examined how respondents wished to engage with NAT-Net. The most frequently selected priorities were connecting and collaborating (103/124; 83.1%), attending networking events (91/124; 73.4%), accessing training opportunities (86/124; 69.4%), and receiving updates or newsletters (86/124; 69.4%). Additional priorities included participation in working groups, access to supporting services, and contributing expertise or training. Responses under “Other” highlighted strategic aspirations such as developing and implementing a national plan for the delivery of NATs and providing technologies that can substitute animal models in drug discovery and biomedical research (Figure 4A).

### Perceived challenges and gaps

To identify priority areas for network action, respondents were asked what challenges or gaps NAT-Net should help address. Among those answering this question (n = 120/124), the highest priorities were the establishment of standards and validation processes (91/120; 75.8%), improved access to resources (89/120; 74.2%), and support for finding collaborators or partners (86/120; 71.7%). Education (66/120; 55.0%) and policy or regulatory advocacy (65/120; 54.2%) were also identified as key areas (Figure 4B).

Free-text responses emphasised the need for a coordinated national strategy, clearer regulatory guidance, improved visibility and coordination of infrastructure, workforce development, and balanced public communication. Additional suggestions included highlighting relevant NAT/NAM funding opportunities, collating, and disseminating information on NAM development and validation, lobbying and reassuring policymakers about the validity of replacement technologies, harmonising and maximising resources in Australia, improving awareness of available technologies, and linking investments to deliver national infrastructure while minimising duplication. Respondents also noted the importance of reinvigorating the 3Rs imperative, retaining skilled experts within NSW, and addressing funding gaps (Figure 4C).

### Exploratory comparisons (hypothesis-generating)

Because the survey used convenience sampling and was geographically skewed toward New South Wales, we removed jurisdiction-based inferential comparisons and report jurisdictional distributions descriptively only. As an exploratory, within-sample analysis (not adjusted for multiple comparisons), respondents engaged in R&D who reported using *in vitro* 3D models were more likely to nominate standards and validation as a key challenge than those not using 3D models (81.8% vs. 56.3%; Fisher’s exact test, p = 0.013). This observation should be interpreted cautiously and treated as hypothesis-generating within the respondent sample only and is not generalisable to the broader Australian NAT ecosystem.

## Discussion

At a high level, Australia’s organisation activities in the non-animal technologies space encompass championing the adoption of animal-free alternatives and advocating for the 3Rs; developing and commercialising human tissue models, organoids, and lab-on-a-chip platforms; advancing *in silico* and *in vitro* methods for safety and efficacy; providing training on cell culture practices and embedding NATs in education; patenting innovative biotechnologies and partnering for scale-up; and offering strategic and economic advisory services. Other roles included computational modelling, biomarker data collection, and ethical animal welfare assessments, as well as transition initiatives for reducing animal use.

### Overview of key findings in an international context

This study provides an initial cross-sectoral snapshot of the stakeholders engaged in the Australian non-animal technologies ecosystem through NAT-Net dissemination pathways, positioning the network as a potential national coordinating body. Respondents spanned universities, medical research institutes, national infrastructure, hospitals, industry, government, advocacy, and the public, demonstrating that NAT activity in Australia is not confined to specialist method-development groups but distributed across many parts of the biomedical and innovation system. NAT-related activities were similarly broad, extending beyond research and development to include education and training, ethics and 3Rs activities, infrastructure provision, commercialisation, funding, policy, and advocacy.

Among respondents involved in research and development, NATs were deployed across a wide range of scientific domains, with strong representation in enabling areas such as stem cell research, drug development and testing, method development and molecular biology/genetics. Technology portfolios were multi-modal: advanced *in vitro* 3D models (including organoids and spheroids), microphysiological systems/organ-on-chip and bioprinting featured prominently alongside 2D systems, *in silico* modelling, *in chemico* assays, tissue explants and patient-based research. Most activity was concentrated at fundamental, discovery and preclinical stages, with relatively few technologies reported as being used in clinical development or regulatory contexts—consistent with international observations that coordinated validation, standardisation and regulatory engagement remain key determinants of broader adoption and should be areas of focus for Australia in building a mature NAT sector. Viewing these development stages as an indicative NAT maturity model provides a useful lens for policy and investment. The skew toward early-stage work suggests that Australia has strong scientific capability and model development expertise, but limited penetration of NATs into later-stage clinical and regulatory use. This mirrors international experience, where bottlenecks are less about inventing new models and more about validating them, embedding them in decision frameworks, and building confidence among regulators, ethics committees and end-users. Framing survey results along this maturity spectrum therefore highlights where targeted effort—such as coordinated validation studies, shared reference data and structured regulator engagement—could have the greatest impact on accelerating NAT adoption. Accordingly, inferential findings are included to inform future investigation rather than to support population-level inference.

The survey also identified substantial supporting infrastructure, with many service providers indicating external access through fee-for-service or collaboration models. Respondents expressed a strong appetite to engage with NAT-Net *via* collaboration, networking, training, and working groups, and they highlighted standards and validation, access to resources, collaboration, education and policy/regulatory engagement as priority challenges a coordinated national network could help address. Together, these findings position NAT-Net as an ecosystem-level complement to method-focused initiatives by capturing not only “what” NATs are being used, but also “who” is involved, “how” capabilities can be accessed, and “where” system-level barriers are felt most acutely. The findings should therefore be interpreted as a network-anchored ecosystem mapping rather than a comprehensive census of all Australian NAT activity. While similar challenges may exist across the broader Australian ecosystem, these observations derive from the participating respondent cohort and require wider national participation to confirm general isability. Accordingly, national-level implications should be considered indicative and hypothesis-generating rather than demonstrative. Consequently, recommendations should be interpreted as guidance for coordinated development rather than evidence of uniform national need. A recent Australian national capability mapping conducted by The Commonwealth Scientific and Industrial Research Organisation (CSIRO, 2023) also reported distributed expertise, infrastructure fragmentation and validation challenges across Australia’s non-animal modelling landscape. While the CSIRO, 2023 survey primarily catalogued technical capabilities, the present study complements this by capturing cross-sector stakeholder perspectives, coordination needs and engagement preferences. Together, these complementary findings indicate that Australia possesses substantial technical capacity but may benefit from structured coordination, visibility and validation pathways to translate capability into routine adoption.

While Australia hosts pockets of world-class expertise and infrastructure in non-animal technologies, there is a notable gap in structured consultation and dedicated funding for the implementation of NATs. Internationally, EMA provides organized regulatory interactions, such as briefing meetings and scientific advice to facilitate the acceptance of NAMs, ensuring they are scientifically sound and integrated into regulatory decision-making processes. Similarly, the NIH, through the Complement-ARIE program, committed approximately $35–40 million annually over a 10-year period to accelerate the development, validation, and regulatory adoption of NAMs through technology development centres, data hubs, and validation networks (National Institutes of Health, 2024). In contrast, Australia does not yet have similarly coordinated, resourced mechanisms to support systematic validation, qualification, and alternative regulatory pathways for NAMs, nor sustained financial investment, despite recent advocacy calling for a national funding stream and a dedicated centre to coordinate NAMs' infrastructure and training. Addressing this gap is critical to ensure that Australia not only maintains scientific excellence but also accelerates the humane, ethical and innovative transition toward 3Rs-compliant methodologies in biomedical research and testing.

### Other challenges for the Australian NAT ecosystem and NAT-Net

Several other challenges emerge that are likely to shape NAT-Net’s trajectory. First, defining the scope of “non-animal technologies” is inherently complex: some stakeholders self-identify strongly with alternatives/NAM language, while others working in enabling fields (e.g., AI/ML, imaging or specific clinical domains) may contribute directly to non-animal approaches through a parallel technology stream without framing their activities as NATs as they are derived from developments in unrelated scientific disciplines. Communicating an inclusive but coherent scope will be critical to ensure NAT-Net captures relevant expertise without diluting its purpose.

Second, geography and governance matter. Australia’s NAT ecosystem is dispersed across a large, federated country with varied institutional cultures, funding arrangements and regulatory responsibilities. The baseline skew toward New South Wales likely reflects NAT-Net’s origins, initial funding and early dissemination pathways; sustained growth will require deliberate strategies to expand participation in underrepresented jurisdictions and sectors to achieve a genuinely national map.

Third, incentives and trust are central to maintaining a living ecosystem map. Participation requires time and ongoing updates from busy stakeholders, and willingness to share information can be shaped by Intellectual Property concerns, competition, and reputational risk. The survey, therefore, focused on high-level capabilities rather than sensitive technical details; as NAT-Net evolves toward more public-facing directories or registries, clear data governance, permissions and terms of use will remain essential. Finally, long-term sustainability including dedicated staff time and resourcing will be needed to maintain mapping, coordinate working groups, deliver training, and sustain engagement with regulators, funders and the public.

### Added value of NAT-Net compared with existing initiatives

A key contribution of this work is the introduction of an ecosystem framing for NATs. NAT-Net emphasises the broader system beyond technical capability, incorporating organisations, people, infrastructure access, engagement preferences, and perceived barriers such as education, ethics, regulation, policy, and communication. This breadth positions NAT-Net as more than a directory; it is a strategic enabler for national coordination. Mapping and updating data generated through this framework can inform national strategy and investment by identifying areas of strength and gaps, guiding shared infrastructure access, and supporting regulators and ethics committees to understand where replacement and reduction may be near-term possibilities. This approach aligns with global efforts such as the International Cooperation on Alternative Test Methods (ICATM), which brings together agencies from the EU, US, Japan, Canada, South Korea, Brazil, and others to harmonise validation and regulatory acceptance of NAMs globally (International Cooperation on Alternative Test Methods, 2024). Similarly, the STEP4NAMs Project, a 4-year EU-funded consortium, is developing strategies to validate NAMs, create a NAM validation handbook, and establish training programs for the pharmaceutical and medical technology sectors (STEP4NAMs Consortium, 2023). By adopting an ecosystem perspective, NAT-Net positions Australia to accelerate innovation, foster regulatory confidence, and strengthen international alignment in the transition toward NATs methodologies.

Importantly, because the mapping tool is embedded within NAT-Net membership processes, it can be updated as the network grows and as technologies and regulatory expectations evolve, creating the potential for a dynamic, living resource that complements method-focused repositories by adding a national, cross-sector ecosystem lens.

## From baseline mapping to ecosystem development: strategic implications for Australia’s NAM/NAT landscape

The priorities below derive from two complementary sources: (i) needs directly identified by survey respondents and (ii) strategic recommendations arising from synthesis of the survey findings with NAT-Net’s coordination mandate and international policy context. Where relevant, long-term measures therefore reflect broader strategic alignment rather than direct survey nomination alone. The baseline findings identify clear, actionable priorities for strengthening Australia’s non-animal technologies ecosystem, particularly in relation to coordination, infrastructure access, validation, training and regulatory engagement.

Short-term priorities (1–2 years):

- Expand participation (particularly in underrepresented jurisdictions, sectors, and technology areas, ensuring a more complete national map.
- Refine NAT categories and maturity descriptors to better capture emerging approaches and align with international terminology.
- Develop a secure, appropriately permissioned directory of national capabilities and infrastructure to support discovery of expertise, services, and collaboration opportunities.
- Establish initial working groups focused on high-demand areas such as standards and validation, regulatory engagement, and training.

Medium term priorities (3–5 years)

- Co-develop validation frameworks, reference datasets and best-practice guidelines with industry working groups (researchers, industry, and regulators) to support wider NAT adoption.
- Build layered training and workforce development programs, including online modules, short courses, and regulator-ethics capacity-building activities.
- Strengthen access pathways to national infrastructure through shared facilities, fee-for-service models and coordinated investment strategies.
- Pilot collaborative case studies demonstrating NAT integration in preclinical decision-making pipelines.

Long-term priorities (5+ years)

- Maintain a dynamic-longitudinal ecosystem map through periodic follow-up surveys and targeted deep-dive analyses.
- Support integration of NATs into regulatory and policy frameworks by providing evidence-based recommendations informed by longitudinal data.
- Facilitate national coordination of strategic investment in shared infrastructure, validation efforts and workforce capacity.
- Promote international alignment by contributing to global NAT initiative, harmonisation activities and cross-border regulatory dialogue (strategic alignment recommendation (policy informed).

## Limitations

Key limitations of this study include: (1) convenience sampling through early NAT-Net dissemination channels likely introduced sampling and geographic bias, with over-representation of New South Wales–based and network-connected organisations and potential self-selection bias toward stakeholders already engaged in non-animal technologies. Accordingly, these data should not be used to infer jurisdiction-level differences in NAT activity or capacity. (2) all data were self-reported and may be subject to misclassification of activities, model types and technology maturity and were not independently validated. (3) the cross-sectional design provides a snapshot at a single time point in a rapidly evolving NAT landscape; follow-up mapping will be required to track changes in capabilities, adoption, and barriers. (4) infrastructure visibility is inherently unequal, and services that are less formalised or not yet linked to NAT-Net may be under-represented in our sample and multiple respondents from the same organisation may contribute clustered perspectives. Furthermore, the open dissemination strategy precluded calculation of a response rate and limits inference regarding population representativeness.

Despite these constraints, the survey offers a useful empirical snapshot to inform NAT-Net’s development and national coordination, investment, and regulatory engagement in non-animal technologies.

## Data Availability

All relevant data, with direct identifiers removed to ensure compliance with privacy legislation, are available from the authors upon reasonable request.
